# Phase I-II study of everolimus and low-dose oral cyclophosphamide in patients with metastatic renal cell cancer

**DOI:** 10.1186/1471-2407-11-505

**Published:** 2011-11-30

**Authors:** Charlotte M Huijts, Saskia J Santegoets, Alfons J van den Eertwegh, Laura S Pijpers, John B Haanen, Tanja D de Gruijl, Henk M Verheul, Hans J van der Vliet

**Affiliations:** 1 Department of Medical Oncology, VU University Medical Center, De Boelelaan 1117, 1081 HV, Amsterdam, The Netherlands; 2 Division of Immunology, The Netherlands Cancer Institute, Plesmanlaan 121, 1066 CX, Amsterdam, The Netherlands

## Abstract

**Background:**

For patients with metastatic renal cell cancer (mRCC) who progressed on vascular endothelial growth factor (VEGF) receptor tyrosine kinase inhibitor therapy, the orally administered mammalian target of rapamycin (mTOR) inhibitor everolimus has been shown to prolong progression free survival. Intriguingly, inhibition of mTOR also promotes expansion of immunosuppressive regulatory T cells (Tregs) that can inhibit anti-tumor immune responses in a clinically relevant way in various tumor types including RCC. This study intends to investigate whether the antitumor efficacy of everolimus can be increased by preventing the detrimental everolimus induced expansion of Tregs using a metronomic schedule of cyclophosphamide.

**Methods/design:**

This phase I-II trial is a national multi-center study of different doses and schedules of low-dose oral cyclophosphamide in combination with a fixed dose of everolimus in patients with mRCC not amenable to or progressive after a VEGF-receptor tyrosine kinase inhibitor containing treatment regimen. In the phase I part of the study the optimal Treg-depleting dose and schedule of metronomic oral cyclophosphamide when given in combination with everolimus will be determined. In the phase II part of the study we will evaluate whether the percentage of patients progression free at 4 months of everolimus treatment can be increased from 50% to 70% by adding metronomic cyclophosphamide (in the dose and schedule determined in the phase I part). In addition to efficacy, we will perform extensive immune monitoring with a focus on the number, phenotype and function of Tregs, evaluate the safety and feasibility of the combination of everolimus and cyclophosphamide, perform monitoring of selected angiogenesis parameters and analyze everolimus and cyclophosphamide drug levels.

**Discussion:**

This phase I-II study is designed to determine whether metronomic cyclophosphamide can be used to counter the mTOR inhibitor everolimus induced Treg expansion in patients with metastatic renal cell carcinoma and increase the antitumor efficacy of everolimus.

**Trial Registration:**

ClinicalTrials.gov Identifier NCT01462214, EudraCT number 2010-024515-13, Netherlands Trial Register number NTR3085.

## Background

Approximately 2% of all adult malignancies are kidney tumors and these account for about 116.000 deaths worldwide per year [1]. Renal cell carcinoma (RCC) is the most common primary tumor arising in the kidney and can be classified into four histological subtypes, i.e. clear cell (60-80%), papillary (10-15%), chromophobe (5-10%) and collecting duct carcinoma (< 1%). Approximately 30% of all patients with RCC has metastatic disease at presentation and ~50% of patients undergoing curative surgery can be expected to experience relapse at distant sites [2,3].

The treatment of metastatic RCC (mRCC) has considerably changed over the last 5 years due to the antitumor efficacy of two groups of targeted agents, namely agents that inhibit vascular endothelial growth factor (VEGF)-signaling pathways and those that inhibit mammalian target of rapamycin (mTOR) [4]. The oral multi-targeted receptor tyrosine kinase inhibitor sunitinib displays a 31% objective response rate in mRCC patients with disease stabilization and an increase in median progression free survival from 5 to 11 months [5]. For patients with mRCC that progressed on VEGF receptor tyrosine kinase inhibitor therapy, the orally administered mTOR inhibitor everolimus was recently shown to prolong progression free survival relative to placebo from 1.9 months to 4.9 months (p < 0.001), providing an important additional therapeutic tool for this patient category [6,7].

As a derivative of rapamycin, everolimus acts as a signal transduction inhibitor that is selective for mTOR. mTOR is a key protein kinase present in all cells which regulates cell growth, proliferation, angiogenesis, and survival. The mTOR pathway also plays an important role in immunoregulation. It critically controls homeostasis and the balance between effector T cells and regulatory T cells [8-11]; inhibition of mTOR has been shown to result in expansion of immunosuppressive regulatory T cells in vitro and in vivo [12-14].

CD4^+^CD25^+ ^regulatory T cells (Tregs) represent a functionally distinct lineage of immunoregulatory T cells crucial for the maintenance of tolerance. Tregs are critically dependent on the X-chromosome encoded FOXP3 gene. Mutations in this gene, that encodes the forkhead/winged-helix family transcriptional repressor Scurfin, cause a fatal human autoimmune disorder called IPEX (immune dysregulation, polyendocrinopathy, enteropathy, X-linked syndrome) [15]. Tregs have been shown to suppress anti-tumor immune responses. In murine models Tregs selectively accumulate in the tumor and locally maintain a microenvironment that suppresses the effector function of tumor-infiltrating cytotoxic lymphocytes. Furthermore, in melanoma Tregs are overrepresented in metastatic lymph nodes where they inhibit the function of infiltrating immune cells. The frequency of Tregs has also been reported to be increased in the circulation and/or tumor microenvironment of patients with various cancers, including RCC [16]. Of note, both the frequency of circulating as well as peritumoral Tregs are associated with adverse outcome in several tumor types [16-18]. Importantly, depletion of Tregs can take the brake off of suppressed immune responses and result in a substantially potentiated antitumor immune response. E.g. Treg depletion can result in the rapid rejection of well-established tumors, can enhance natural tumor immunosurveillance and can enhance vaccine efficacy of weakly immunogenic melanoma cells [19-21]. Notably, metronomic administration of cyclophosphamide has consistently been reported to induce Treg depletion in both murine and human studies and to result in a restoration of T and NK effector functions [19,22-24].

Based on these data, the aim of the present study is to evaluate whether metronomic cyclophosphamide can be used to counter the mTOR inhibitor everolimus induced Treg expansion in patients with metastatic renal cell carcinoma and increase the antitumor efficacy of everolimus.

## Methods/design

**Figure 1:**
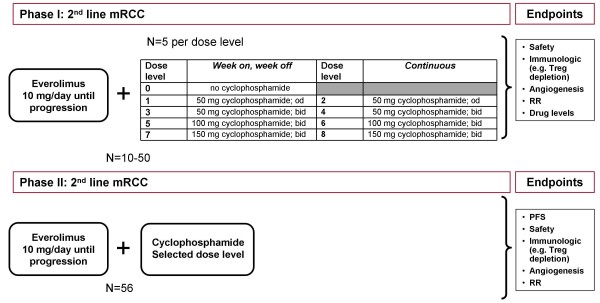
**Trial design**. Patients will be enrolled in cohorts of 5 per dose level. The first 5 patients enrolled will be assigned to dose level 0 in order to assess immune and anti-angiogenic effects of everolimus monotherapy. In subsequent dose levels the cyclophosphamide dose will be increased up to a maximum of 150 mg cyclophosphamide twice daily. The dose level showing the most selective depletion of Tregs will be selected for phase II. Endpoints for the phase I part are safety, immunologic and angiogenesis parameters, response rate and drug levels. In the phase II part of the study patients will receive 10 mg everolimus per day in combination with the optimal Treg depleting dose cyclophosphamide. Endpoints for the phase II part are progression free survival, safety, immunologic and angiogenesis parameters and response rate.

In the phase I part of the study we will determine the optimal Treg depleting dose and schedule of metronomic oral cyclophosphamide when given in combination with a fixed dose of everolimus. Patients will be enrolled in cohorts of 5 per dose level (see Figure 1). The dose level showing the most selective depletion of Tregs will be selected for phase II. In the phase II part of the study we will evaluate whether the number of patients who are progression free at 4 months can be increased from 50% [6,7] to 70% by adding metronomic cyclophosphamide to everolimus.

### Primary objectives

#### Phase I

Assessment of the recommended dosing and schedule for metronomic cyclophosphamide with respect to the optimal selective induction of CD4^+^CD25^+ ^regulatory T cell depletion, when administered in combination with fixed dose (10 mg) oral everolimus in patients with mRCC.

#### Phase II

To investigate the proportion of patients with mRCC receiving everolimus and metronomic cyclophosphamide that is progression free at 4 months.

#### Phase I and II

To determine the safety and tolerability of the everolimus-cyclophosphamide combination.

### Secondary objectives

- To assess the response rate, time to progression and overall survival of the combination of metronomic cyclophosphamide and fixed dose oral everolimus.

- Assessment of the immunological effects of combining metronomic cyclophosphamide with everolimus focusing on its impact on the number and function of circulating and tumor-infiltrating CD4^+^CD25^+ ^regulatory T cells.

- Assessment of the effect of the combination of metronomic cyclophosphamide and everolimus on selected angiogenesis parameters.

- To assess the effect of cyclophosphamide on everolimus drug levels.

### Design

This phase I-II trial is a national multi-center dose-escalation study of low-dose oral cyclophosphamide in combination with a fixed dose of everolimus in patients with mRCC not amenable to, or progressive after, a VEGF-receptor tyrosine kinase inhibitor containing treatment regimen.

### Setting

The study is initiated by the department of medical oncology of the VU University Medical Center and will be conducted within the context of the Netherlands Working Group on Immunotherapy of Oncology (WIN-O) with a present participation of 13 hospitals.

### Eligibility criteria

#### Inclusion criteria to be eligible for the trial

1. Signed informed consent.

2. Age ≥ 18 years.

3. Histological or cytological confirmed clear-cell mRCC with progressive disease and not amenable to, or progressive on, or within 6 months of stopping treatment with a VEGF receptor tyrosine kinase inhibitor (sunitinib (or pazopanib) ± sorafenib).

4. No other current malignant disease, except for basal cell carcinoma of the skin.

5. WHO performance status 0-2.

6. Life expectancy of at least 12 weeks.

7. Adequate hematologic function: ANC ≥ 1.5 × 109/L, platelets ≥ 100 × 109/L, Hb ≥ 6.0 mmol/L.

8. Adequate hepatic function: serum bilirubin ≤ 1.5 × ULN, ALT and AST ≤ 2.5 × ULN (or ≤ 5 times ULN if liver metastases are present).

9. Adequate renal function: calculated creatinine clearance ≥ 50 ml/min.

10. Measurable or evaluable disease as defined by RECIST 1.1.

11. Patients with reproductive potential must use effective contraception, negative pregnancy test for female patients.

12. Able to receive oral medication.

13. Prior therapy with cytokines (i.e. IL-2, interferon) and/or VEGF-ligand inhibitors (i.e. bevacizumab) is permitted.

14. Patients with brain metastases are eligible if they have been stable for at least two months post-radiation therapy or surgery.

#### Exclusion criteria

1. Currently receiving chemotherapy, immunotherapy, or radiotherapy, or having received either one of these ≤ 4 weeks prior to visit 1. The wash-out period for sunitinib or sorafenib is at least 2 weeks.

2. Known human immunodeficiency virus (HIV) or other major immunodeficiency.

3. Treatment with immunosuppressive agents within 3 weeks of study entry, except for low dose corticosteroids.

4. Active bleeding diathesis or oral anti-vitamin K medication.

5. Patients with untreated CNS metastases with clinical symptoms or who have received treatment for CNS metastases within 2 months of study entry.

6. Active infection or serious intercurrent illness, except asymptomatic bacteriuria.

7. Presence of unstable angina, recent myocardial infarction (within the previous 6 months), or use of ongoing maintenance therapy for life-threatening ventricular arrhythmia.

8. Macroscopic hematuria.

9. Prior therapy with mTOR inhibitors.

10. Known hypersensitivity to everolimus or other rapamycins (sirolimus/temsirolimus) or to its excipients.

11. Pregnant or nursing women or women of childbearing potential not utilizing an effective contraceptive method. Men with partners of childbearing potential not using an effective method of contraception. (Use of effective contraceptives must continue for 3 months after the last dose of everolimus).

12. Presence of any significant central nervous system or psychiatric disorder(s) that would hamper the patient's compliance.

13. Uncontrolled diabetes as defined by fasting serum glucose > 2 ULN, severely impaired lung function.

14. Cirrhosis/chronic active hepatitis/chronic persistent hepatitis, history of hepatitis B or C virus infection.

15. Drug or alcohol abuse.

16. Any other major illness that, in the investigator's judgment, substantially increases the risk associated with the subject's participation in the study.

### Ethics

The study will be conducted in accordance with ethical principles that have their origin in the Declaration of Helsinki and are consistent with International Conference on Harmonisation (ICH) Guidelines for Good Clinical Practice. This protocol has been submitted and approved by the Medical Ethical Committee of the VU University Medical Center, Amsterdam, the Netherlands and the Central Committee on Research Involving Human Subjects (CCMO). Approval of independent medical ethics committees of participating hospitals is required before local initiation of the study protocol. Oral and written informed consent is obtained from all patients prior to trial participation.

### Safety

All adverse events encountered during the clinical study will be reported on case report forms (CRF). In case of a serious adverse event (SAE), the investigator has a legal requirement to inform the central data management office within 24 hr through the web portal *ToetsingOnline*. All SAEs must also be reported on the Adverse Event page of the CRF.

The principal investigators are responsible for the management of the safety reporting according to local regulations and guidelines. The study drug license holder will be provided with copies of all report submissions to regulatory authorities and to the ethical committee that has approved the study by the principal investigators. All reported (serious) adverse events will be discussed by an independent data monitoring committee. Novartis is responsible to ensure that the latest investigator's brochure is used as the source document for determining the expectedness of a SAE.

### Monitoring

The study progress, safety data and data quality will be monitored by an Independent Data (and safety) Monitoring Board (IDMB), which will be independent of the trial organizers. Safety analysis will be performed on a regular basis and the IDMB will report their findings to the principal investigators. Once a year throughout the clinical trial, the principal investigators will submit a safety report to the accredited METC, including a list of all suspected (unexpected or expected) serious adverse reactions, along with an aggregated summary table of all reported serious adverse reactions. In addition, a complete safety analysis and an evaluation of the balance between the efficacy and the harmfulness of the medicine under investigation will be submitted.

#### Data quality assurance

Data management and trial coordination will be performed via the use of OpenClinica^®^. This open source web-based software platform for clinical trial data is compatible with the cancer Biomedical Informatics Grid (caBIG^®^) from the U.S. National Cancer Institute to manage clinical research data in multi-site studies.

### Interim analysis

In the phase II part of the study an interim analysis (IA) will be performed after treatment of 24 patients that reached 4-month progression free survival (PFS) or that progressed within the first 4 months. If the number of patients progression free at 4 months is < 12 the study will be terminated due to lack of efficacy. In addition, the number of patients with combination treatment related grade ≥ 3 toxicity will be analyzed and if this number is 12 or higher, the study will be terminated because of unacceptable toxicity.

Due to the time dependent nature of the outcome of interest, it is possible that there will not be sufficient evidence to either terminate or continue the trial at the time of enrollment of the 24th individual. In that case, the PFS rate at 4 months for the previously started patients will determine whether or not to halt the trial due to lack of efficacy. A decision must be made whether or not to halt enrollment until these 24 patients have been followed for 4 months or to continue enrollment and risk completing enrollment for the trial before the interim analysis is made. If only 3 of the first 24 patients have a response, then enrollment will be halted until the 4-month progression free survival interim analysis is completed.

### Statistical Analysis

In the phase I part of the study, patients are defined as evaluable if they have completed a minimum of 2 weeks of combination therapy (i.e. allowing monitoring of immune effects at t = 2 weeks). Statistical analysis in the phase II part will be performed according to the intention-to-treat principle. All patients in this part of the study that have taken their study medication on at least one occasion are considered evaluable patients.

The response rate will be determined as the proportion of treated patients who had a partial or complete response. Time to progression (TTP) and overall survival will be constructed by the use of Kaplan-Meier curves. TTP will be measured from enrollment and preferably be defined based on CT-scan imaging based on Response Criteria according to RECIST (version 1.1) or alternatively based on clinical evaluation. For TTP analysis death is considered as progressive disease. Median TTP and overall survival will be calculated along with 95% confidence intervals. Statistical analyses of changes in immune and angiogenesis parameters and drug levels will be performed using paired Student t tests or Wilcoxon matched pairs tests as appropriate. P < 0.05 will be considered statistically significant.

The sample size in the Phase I part of the study is expected to be 10-50 patients. Patients will be enrolled in cohorts of 5 per dose level. The first 5 patients will be assigned to dose level 0, to assess the effects of everolimus monotherapy. Subsequently, 8 combination therapy dosing levels are planned (see Figure 1). At the final dose level recommended for the phase II study a minimum of 10 patients will be treated. For phase II, a maximum of 56 patients will be included. The sample size for this part is determined based upon a Bryant Day Phase II clinical trial design, taking into account both activity as well as toxicity. Statistical calculation parameters for the phase II part of this study are: Probability of Accepting Poor Response (α-r) = 0.1, Probability of Accepting Toxic Drug (α-t) = 0.1, Probability of Rejecting Good Drug (β) = 0.1, Unacceptable Response Probability (Pr0) = 0.5. Acceptable Response Probability (Pr1) = 0.7, Unacceptable Non-toxicity Probability (Pt0) = 0.5, Acceptable Non-toxicity Probability (Pt1) = 0.7, Early termination probability = Poor Response and Excessive Toxicity = 0.82, Poor Response and Acceptable Toxicity = 0.59, Good Response and Excessive Toxicity = 0.59 and Good Response and Acceptable Toxicity = 0.06. Taking these parameters into account, the limit for 1st stage rejecting of the drug due to inadequate response at interim analysis will be ≤ 12 patients without being progression free at 4 months. The upper limit for 2^nd ^stage (when total inclusion is completed) rejecting of the drug due to inadequate response is ≤ 32 patients without being progression free at 4 months. At least 32 patients should be without grade ≥ 3 toxicity due to therapy, otherwise the combination therapy will be rejected due to excessive toxicity.

### Follow up

#### Assessments during study treatment phase

- Baseline evaluations are to be conducted within 2 weeks prior to the start of protocol therapy, and comprise medical history, physical examination, toxicity assessment, hematology, serum chemistry, immune and angiogenesis monitoring, ECG, tumor biopsy (not mandatory) and, in case of female participants, urine HCG. Abdominal and chest CT scans must be done within 4 weeks prior to start of therapy.

- Patient visits will be scheduled at 2, 4 and 8 weeks and subsequently 4-weekly. At each visit medical history, physical examination, toxicity assessment, hematology and serum chemistry will be performed.

- Immune monitoring on CD4^+^CD25^+^FOXP3^+ ^regulatory T cells, conventional T cell subsets, NK cells, invariant CD1d-restricted NKT cells, dendritic cell subsets, myeloid-derived suppressor cells and cytokine levels will be performed at baseline and subsequently at 2, 4, and 8 weeks after the start of the study treatment period.

- Angiogenesis monitoring on thrombospondin 1 (TSP-1), VEGF, circulating numbers of platelets and hematopoietic progenitor cells (HPCs) will be performed at baseline, week 4 and 8.

- Everolimus and cyclophosphamide drug levels will be measured during the phase I part of the study at week 4.

- If patients agreed on a tumor biopsy, a second biopsy will be obtained at week 4.

- CT images will be required at fixed time-points; baseline, 8 and 16 weeks, and thereafter every 8 weeks during treatment until progressive disease.

- Adverse events will be reported during the study treatment phase. Medically significant adverse events considered related to the investigational product by the investigator will be followed until resolved or considered stable.

#### Assessments during follow up phase

- At the end of study treatment, medical history and toxicity will be assessed, and physical examination, hematology, serum chemistry, immune and angiogenesis monitoring, abdominal and chest CT scans will be performed.

- Follow up will last till progressive disease and/or death of the patient.

### Study medication

#### Everolimus

Everolimus is a selective mTOR signal transduction inhibitor. It exerts its activity through high affinity interaction with an intracellular receptor protein, the immunophilin FKBP12. The FKBP12/everolimus complex subsequently interacts with the mTOR protein kinase, inhibiting downstream signaling events. mTOR is mainly activated via the PI3-kinase pathway through AKT/PKB and the tuberous sclerosis complex (TSC1/2). mTOR functions as a sensor of mitogens, growth factors and energy and nutrient levels, facilitating cell-cycle progression from G1-S phase in appropriate growth conditions. This pathway is also involved in the production of pro-angiogenic factors (i.e. VEGF) and stimulation of endothelial cell growth and proliferation. mTOR regulates protein translation through inactivating eukaryotic initiation factor 4E binding proteins and activating the 40S ribosomal S6 kinases, including the HIF-1 proteins [25].

Everolimus has been investigated in various indications, namely solid organ transplantation, hematologic and non-hematologic malignancies, and rheumatoid arthritis. Based on its potential to act directly on tumor cells by inhibiting tumor cell growth and proliferation and indirectly by inhibiting angiogenesis (via inhibition of tumor cell VEGF production and VEGF-induced proliferation of endothelial cells), everolimus was also investigated as an anticancer agent.

Everolimus has been evaluated clinically in patients with mRCC who failed a previous VEGF receptor tyrosine kinase inhibitor treatment and with several other solid malignancies in investigator sponsored studies. Daily doses of 5 and 10 mg have shown target inhibition with satisfactory tolerability in most patients. In general, everolimus is well tolerated and the safety profile is considered favorable and distinct from that of traditional chemotherapy [6,7].

#### Cyclophosphamide

Cyclophosphamide is an alkylating agent that mediates crosslinking of DNA. It is frequently used in high doses in the treatment of several malignancies [26] and induces improved immune responses both in murine and human studies, when used in low doses due to induction of Treg depletion [19,22-24]. Furthermore, depletion of Tregs using metronomic oral cyclophosphamide restores T and NK cell effector functions in end stage cancer patients [22] and anticancer immunity and vaccine efficacy in multiple murine models [19,27].

Metronomic cyclophosphamide has also been reported to inhibit tumor angiogenesis, e.g. by reducing the mobilization and viability of HPCs and by inducing sustained apoptosis of endothelial cells within the vascular tumor bed [27-32]. TSP-1, a potent and endothelial cell specific inhibitor of angiogenesis, appears to be an important mediator of this process as the antiangiogenic and antitumor activity of metronomic cyclophosphamide was abolished in TSP-1 null mice [31]. In elderly breast cancer patients the addition of metronomic cyclophosphamide to letrozole resulted in lower levels of VEGF in the tumor [33].

In the present study, cyclophosphamide will be administered orally in the dose determined by the dose level to which the patient is assigned. As cyclophosphamide requires 4-hydroxylation by cytochrome p450s including CYP3A4, and everolimus is mainly metabolized by CYP3A4 in the liver and to some extent in the intestinal wall, there is a potential interaction between the two drugs [34]. Therefore, everolimus and cyclophosphamide drug levels will be determined during this study.

### Treatment program

#### Phase I

Patients will be enrolled in cohorts of 5 per dose level. The first 5 patients enrolled will be assigned to dose level 0 in order to assess immune and anti-angiogenic effects of everolimus monotherapy. The second 5 patients enrolled will be assigned to dose level 1. If there are ≤ 1 dose-limiting toxicities (DLTs) experienced by the first 5 patients in a cohort during the first 28 days after the first study treatment, patients will be entered in the next dose level. Entry of patients into the expansion cohort will not occur until at least 28 days after the last patient in the escalation phase received his/her first study treatment. At the final dose level recommended for the phase II study a minimum of 10 patients will be treated. Intra-individual dose escalation is not permitted in order to allow determination of any possible cumulative effect and to evaluate the inherent toxicity of the regimen at any given dose level. The 10 patients that are being treated at the final dose level in the phase I part will be included in the subsequent phase II analyses.

In case no depletion of Tregs is observed during the phase I part of the study, we consider the absence of an increase in Tregs as the minimally acceptable outcome for proceeding to the phase II part of the study.

#### Phase II

Up to 56 patients will be treated at the dose level that has been selected based on its capacity to most selectively deplete circulating Treg levels in the phase I part of the study. Treatment will be continued until disease progression and/or death of the patient. In case of progressive disease, the choice of subsequent therapy is at the discretion of the investigator.

## Discussion

The mTOR inhibitor everolimus prolongs PFS in patients with mRCC from 1.9 months to 4.9 months relative to placebo [6,7]. Although it thereby provides an important therapeutic tool for this patient category, mTOR inhibitors have also been shown to result in the expansion of immunosuppressive Tregs. Tregs are able to suppress antitumor immune responses in a clinically relevant way and therefore we hypothesize that depletion of Tregs can enhance the antitumor efficacy of everolimus. Cyclophosphamide has consistently been reported to induce Treg depletion, in both murine and human studies, when used in low doses [19,22-24].

This phase I-II study is designed to a) obtain the optimal Treg depleting dose of metronomic cyclophosphamide when combined with the mTOR inhibitor everolimus in patients with mRCC and b) to determine whether this combined treatment results in enhanced antitumor efficacy of everolimus. The study intends to increase progression free survival at 4 months from 50% to 70% by adding metronomic cyclophosphamide to everolimus.

## Competing interests

The authors declare that they have no competing interests.

The study is partly funded by a grant from Novartis Oncology Netherlands.

Novartis has had no part in study design, data collection, analysis, interpretation, the writing of the manuscript, or the decision to submit for publication.

## Authors' contributions

CMH and HJV drafted the manuscript. HJV wrote the original protocol for the study. All authors read and approved the final manuscript.

## Pre-publication history

The pre-publication history for this paper can be accessed here:

http://www.biomedcentral.com/1471-2407/11/505/prepub
